# Variables that influence *BRAF* mutation probability: A next-generation sequencing, non-interventional investigation of *BRAFV600* mutation status in melanoma

**DOI:** 10.1371/journal.pone.0188602

**Published:** 2017-11-27

**Authors:** Maria Rita Gaiser, Alexander Skorokhod, Diana Gransheier, Benjamin Weide, Winfried Koch, Birgit Schif, Alexander Enk, Claus Garbe, Jürgen Bauer

**Affiliations:** 1 Department of Dermatology, Heidelberg University Hospital, Heidelberg, Germany; 2 Department of Dermatology, Venereology and Allergology, University Medical Center Mannheim, Ruprecht-Karl University of Heidelberg, Mannheim, Germany; 3 Department of Dermatology, University Medical Center Tübingen, Tübingen, Germany; 4 BDS Koch, Schwetzingen, Germany; 5 Roche Pharma AG, Grenzach-Wyhlen, Germany; Universidade de Sao Paulo, BRAZIL

## Abstract

**Background:**

The incidence of melanoma, particularly in older patients, has steadily increased over the past few decades. Activating mutations of *BRAF*, the majority occurring in *BRAFV600*, are frequently detected in melanoma; however, the prognostic significance remains unclear. This study aimed to define the probability and distribution of *BRAFV600* mutations, and the clinico-pathological factors that may affect *BRAF* mutation status, in patients with advanced melanoma using next-generation sequencing.

**Materials and methods:**

This was a non-interventional, retrospective study of *BRAF* mutation testing at two German centers, in Heidelberg and Tübingen. Archival tumor samples from patients with histologically confirmed melanoma (stage IIIB, IIIC, IV) were analyzed using PCR amplification and deep sequencing. Clinical, histological, and mutation data were collected. The statistical influence of patient- and tumor-related characteristics on *BRAFV600* mutation status was assessed using multiple logistic regression (MLR) and a prediction profiler.

**Results:**

*BRAFV600* mutation status was assessed in 453 samples. Mutations were detected in 57.6% of patients (n = 261), with 48.1% (n = 102) at the Heidelberg site and 66.0% (n = 159) at the Tübingen site. The decreasing influence of increasing age on mutation probability was quantified. A main effects MLR model identified age (p = 0.0001), center (p = 0.0004), and melanoma subtype (p = 0.014) as significantly influencing *BRAFV600* mutation probability; ultraviolet (UV) exposure showed a statistical trend (p = 0.1419). An interaction model of age versus other variables showed that center (p<0.0001) and melanoma subtype (p = 0.0038) significantly influenced *BRAF* mutation probability; age had a statistically significant effect only as part of an interaction with both UV exposure (p = 0.0110) and melanoma subtype (p = 0.0134).

**Conclusions:**

This exploratory study highlights that testing center, melanoma subtype, and age in combination with UV exposure and melanoma subtype significantly influence *BRAFV600* mutation probability in patients with melanoma. Further validation of this model, in terms of reproducibility and broader relevance, is required.

## Introduction

The incidence of melanoma has steadily increased over the past few decades, particularly in older age groups (>50 years of age) [1]. In 2012, more than 232,000 new cases were diagnosed and approximately 55,500 deaths were reported worldwide [2]. Risk factors for the development of melanoma include high and/or intermittent exposure to ultraviolet (UV) radiation, multiple dysplastic nevi or moles, a family history of melanoma, and skin type (light skin color, light eye color, or light hair color) [3, 4].

The mitogen-activated protein kinase (MAPK) signaling pathway, a key regulator of cell proliferation, differentiation, migration, and apoptosis, has been implicated in the progression of melanoma [5]. The RAF kinase, BRAF, plays an important role in MAPK signaling, and activating mutations of the *BRAF* gene are detected in 48–69% of melanoma cases [6–9]. The majority of *BRAF* mutations in melanoma occur at V600: 66–94% of mutations occur in codon 600 and involve a valine-to-glutamine substitution (V600E) [6, 10, 11]; 6–28.6% of mutations involve lysine substitutions (V600K) [10, 11]; and 2.3–14.7% of mutations involve aspartic acid or arginine substitutions (V600D or V600R, respectively) [10].

Several studies have investigated the prognostic role of *BRAF* mutations in melanoma, but it remains unclear. A multivariate analysis of *BRAF* mutations in patients with metastatic melanoma (N = 109) did not identify *BRAF* mutation as an independent prognostic indicator of overall survival (OS) [12]. In contrast, a retrospective stage III study of patients with cutaneous melanoma (N = 105) found that median OS and distant metastasis-free survival were significantly lower in those with *BRAFV600* mutations compared with wild-type *BRAFV600*, a reduction of 1.4 years and 26.1%, respectively (p<0.01) [13]. Similarly, in primary melanoma, patients with high expression of BRAF protein had significantly worse OS and disease-specific 5-year survival (n = 370) [14]. However, in another study (N = 437), there was a trend towards reduced distant metastasis-free survival in patients with *BRAF* mutations (p = 0.061), but no difference in OS (p = 0.119) [15].

*BRAF*-targeted kinase inhibitors have become increasingly important in melanoma treatment. For example, vemurafenib (Zelboraf^®^, F. Hoffmann-La Roche Ltd, Basel, Switzerland), a potent inhibitor of BRAF, significantly improved OS and progression-free survival (PFS), compared with dacarbazine, in patients with previously untreated metastatic melanoma harboring a *BRAFV600E* mutation [16]. Dabrafenib (Tafinlar^®^, Novartis, Basel, Switzerland), a reversible inhibitor of *BRAFV600E*, significantly improved PFS in patients with previously untreated stage III melanoma compared with dacarbazine [17].

Routine pre-treatment mutation screening is recommended in patients with advanced disease to determine the suitability of BRAF-targeted therapy [18, 19]. In Europe and the USA, treatment options for first- or second-line treatment of *BRAF*-mutated metastatic melanoma include BRAF/MEK inhibitor combinations [18, 19]. Further knowledge of *BRAFV600* mutations in melanoma may allow prediction of mutation probabilities, and would be beneficial in cases where time for treatment is limited or where tumor tissue is not available. In this retrospective study, we aimed to define the probability and distribution of *BRAFV600* mutations in patients with advanced melanoma using next-generation sequencing (NGS) from two Departments of Dermatology in Germany. We also investigated the relationship between certain clinico-pathological factors and the *BRAF* mutation status.

## Materials and methods

### Ethics statement

All patients from the Heidelberg site provided their written informed consent; direct consent was not required from the Tübingen site for samples stored for >5 years. This study was conducted in accordance with the Declaration of Helsinki and approved by the local ethical committees of Hauttumorzentrum Heidelberg (approval reference: S-091/2011) and the University Medical Center Tübingen (approval reference: 413/2012B02).

### Study design

This was a non-interventional, retrospective study of *BRAF* mutation testing at two centers in Germany (University Hospital Heidelberg and the Department of Dermatology, University Medical Center Tübingen). Archival tumor samples were collected retrospectively and sequentially, from patients with histologically confirmed melanoma (stage IIIB, IIIC, IV) and were not preselected. Data were collected on mutational, clinical, and histological findings, including the *BRAF* mutation testing result, patient characteristics (age, gender, tumor stage, ulceration, TNM classification, melanoma subtype, and UV exposure) and the origin of the sample (location of primary tumor or metastasis). UV exposure was determined according to the location of melanoma on the body. Patients characterized as not having UV-exposed melanoma presented with melanoma in areas not routinely exposed to sunlight, e.g. on the back or upper leg; patients characterized as UV-exposed had melanoma on their extremities. *BRAFV600* mutation status was recorded for each center individually and across both centers. The influence of patient- and tumor-related characteristics on *BRAFV600* mutation status was estimated.

### Polymerase chain reaction (PCR) amplification, NGS, and sequence analysis

DNA extraction from tumor tissue was performed using either a QIAamp DNA formalin-fixed paraffin-embedded (FFPE) Tissue Kit (Heidelberg site; QIAGEN, Hilden, Germany) or the cobas^®^ DNA Sample Preparation Kit (Tübingen site; Roche Molecular Diagnostics, Pleasanton, CA, USA). 10 μm sections (seven at the Heidelberg site and as many as required at the Tübingen site) were cut from formalin-fixed paraffin-embedded tumor blocks for DNA extraction. Additional slides were cut directly before (both sites) and after (Heidelberg site) those for DNA extraction for standard hematoxylin and eosin staining. FFPE tissue blocks with >50% tumor cell content, based on routine hematoxylin and eosin staining examined by board-certified pathologists and histopathologists, and sufficient tissue volume to allow successful DNA isolation only, were considered for further processing and analysis. Tissue material was scratched directly from the slides using sterile razor blades and pooled. Approximately 1–2 μg of DNA was isolated from each tumor block. DNA quality checks were performed by PCR, using a QuBit^®^ fluorometer (Heidelberg site; ThermoFisher Scientific, Waltham, MA, USA) or per an internal Roche Pharma AG protocol (Tübingen site).

Following DNA extraction and quantification, DNA from patients’ biopsies was amplified by PCR and deep-sequenced using the 454 GS Junior System (Roche Applied Sciences, Penzberg, Germany) at both sites, with a cutoff of >1%. The 454 GS Junior System technology is derived from the technological convergence of emulsion PCR and pyrosequencing. PCR reactions were performed using fusion primers containing genome-specific sequences, along with one of 34 distinct 10 bp multiplex identifier sequences, which were used to differentiate samples run together on the same plate, and sequencing adapters specially devised for exon 15 of the *BRAF* gene. Sequencing amplicons were 187 bp (both sites) and 220 bp (Heidelberg site probes only). PCR products were visualized on agarose gel and purified using Ampure-XP DNA-binding paramagnetic beads (Beckman Coulter). Samples were then diluted, pooled, and subjected to emulsion PCR (emPCR). Following emPCR, captured beads with bound DNA were enriched and used for massively parallel pyrosequencing. Only sequencing reads with a minimum mapping quality of 20 and bases with a minimum base quality of 13 (phred score) were considered, resulting in a sensitivity cutoff of >1%. All DNA probes were validated for *BRAF* mutations by alternative sequencing methods including Sanger sequencing (Tübingen probes) or pyrosequencing (Heidelberg probes).

### Statistics

The main objectives of the study were to identify patient-, melanoma-, or center-related characteristics that influence the melanoma mutation probability and probabilities of mutation type; to adapt the previously generated *BRAF* non-interventional protocol statistical model to predict the melanoma status for individual patients and centers; and to assess whether the mutation probabilities of the two centers differed after adjustment for the other relevant covariates.

All clinical data were analyzed descriptively using summary statistics, confidence intervals, and graphical methods. Variables considered for inclusion in the model were patient age (continuous or grouped), gender, sample origin (primary melanoma or metastasis), location of sample (grouped as extremities, trunk, head and neck, other), ulceration, tumor stage, TNM classification, melanoma subtype, and UV exposure. The statistical influence of these patient-, melanoma-, or lab-related procedures and characteristics on *BRAFV600* mutation status was assessed using multiple logistic regression (MLR). This fitted the probabilities of the two response levels of the dependent variable, melanoma mutation (yes/no), using a logistic function of the independent variables. Proceeding from a model developed during a previous *BRAF* biomarker project [20], and taking the results of initial univariate exploration into account, a stepwise mixed strategy of excluding and including relevant variables was conducted. During this process, special care was required to avoid unstable models in view of the relatively small sample size of this study. Initially, main effect models were investigated by including the previously indicated covariates only; during a second step, interaction terms between variables were added and a new interaction model developed and presented. All model-based p values were determined from Likelihood-Ratio Chi-Square F statistics and adjusted for the influence of all other independent variables in the same model. The F Ratio was the ratio of the mean squared for the effect divided by the mean squared for error. A prediction profiler was used to visualize the relationship between *BRAFV600* mutation status and the other covariates and their interactions. Additional exploratory analyses were performed as warranted by the data. All analyses were carried out using SAS JMP V12.2.0.

## Results

### Patients

**Fig 1:**
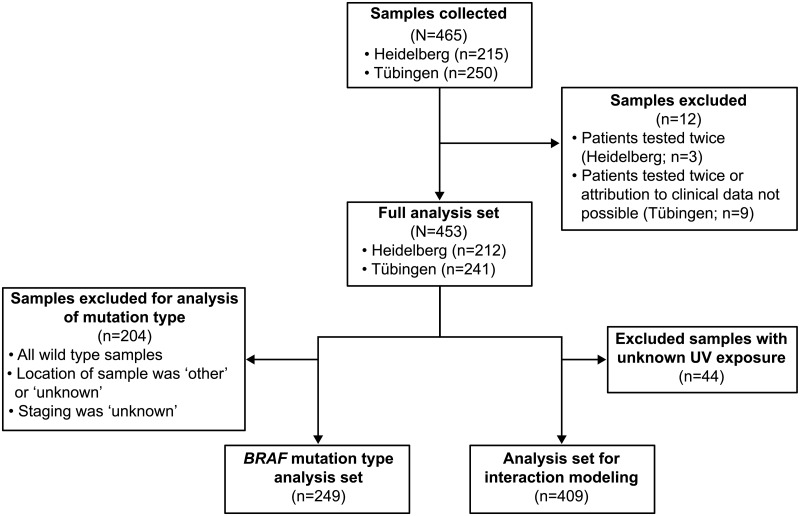
Flow diagram of main analysis sets. UV, ultraviolet.

*BRAFV600* mutation status was determined in 453 samples with stage IIIB, IIIC or IV melanoma using NGS (Heidelberg site: from 1995–2012; Tübingen site: from 1997–2012) [Fig 1].

46.8% of samples (n = 212) were from the Heidelberg site and 53.2% (n = 241) were from the Tübingen site [Table 1], with *BRAFV600* mutations detected in 57.6% of patients. Overall, 48.1% (n = 102) of *BRAFV600* mutations were detected in patients from the Heidelberg site, with 66.0% (n = 159) from patients at the Tübingen site. *BRAFV600* mutations were detected as *BRAFV600E* (44.4%; n = 201), *BRAFV600E2* (3.1%; n = 14), *BRAFV600K* (3.5%; n = 16), *BRAFV600E;K601I* (3.1%; n = 14) and others (Heidelberg site: 1x S607P, 1x S605S, 1x S605N, 1x L597Q, 1x K601R, 2x K601E; 3.3% [n = 7]; Tübingen site: 3x V600M, 1x V600G; 2x V600D, 3x K601E; 3.7% [n = 9] [Table 1 and S1 Table]). 31.1% of patients at the Heidelberg site (n = 66) and 56.0% of patients at the Tübingen site (n = 121) had *BRAFV600E* mutations. The *BRAFV600E2;K601I* tandem mutation was detected only in patients from the Heidelberg site (6.6%; n = 14).

**Table 1:** Patient characteristics and *BRAF* mutation status.

	All (N = 453)	Heidelberg (n = 212)	Tübingen (n = 241)
Age, mean (years)	60.9	60.8	60.9
*BRAF* mutation, n (%)			
Yes	261 (57.6)	102 (48.1)	159 (66.0)
No	192 (42.4)	110 (51.9)	82 (34.0)
*BRAF* mutation type, n (%)			
WT	192 (42.4)	110 (51.9)	82 (34.0)
V600E	201 (44.4)	66 (31.1)	135 (56.0)
V600E2	14 (3.1)	11 (5.2)	3 (1.2)
V600K	16 (3.5)	4 (1.9)	12 (5.0)
V600E2;K601I	14 (3.1)	14 (6.6)	0
Others^a^	16 (3.5)	7 (3.3)	9 (3.7)

^a^ Others include: L597Q, V600M, V600G, V600D, K601R, K601E, S605S, S605N, S607P

**Fig 2:**
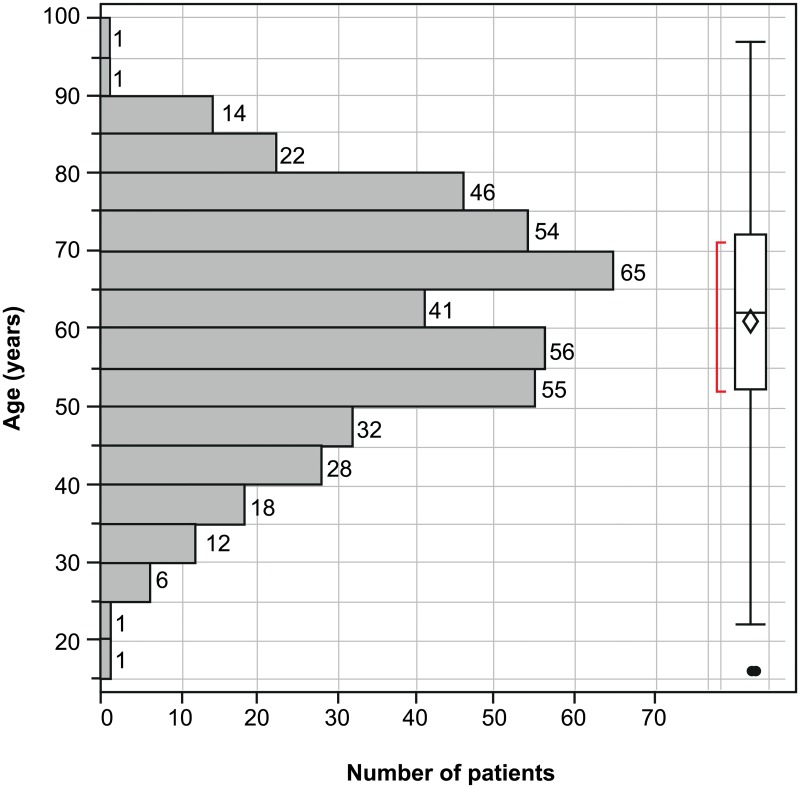
Distribution of patient age at time of sampling.

Overall, the mean age of participants was comparable for both sites (60.8 years for Heidelberg and 60.9 years for Tübingen) and there was no major statistical difference in age distribution [Table 1 and Fig 2]. However, the Heidelberg site had fewer patients in the 60–65 years age group than the Tübingen site.

More than one-third of patients had superficial spreading melanoma (SSM; 44.5%), with nodular melanoma (NM; 22.5%), acral lentiginous melanoma (ALM; 13.2%) and lentigo maligna melanoma (LMM; 9.5%) also detected; 10.3% of cases were not classified [Table 2]. Forty-four samples where the melanoma subtype and UV exposure were unknown were not included in Table 2. The majority of melanoma samples had developed on non-UV-exposed areas (83.4%).

**Table 2:** Melanoma subtype by UV exposure.

Melanoma subtype, n (%)	All (N = 409^a^)	Not UV-exposed (n = 341)	UV-exposed (n = 68)
ALM	54	46 (85.2)	8 (14.8)
LMM	39	8 (20.5)	31 (79.5)
NM	92	81 (88.0)	11 (12.0)
SSM	182	176 (96.7)	6 (3.3)
Unknown	42	30 (71.4)	12 (28.6)

^a^ Forty-four samples where both the melanoma subtype and UV exposure were unknown were omitted from this table. ALM, acral lentiginous melanoma; LMM, lentigo maligna melanoma; NM, nodular melanoma; SSM, superficial spreading melanoma, UV, ultraviolet.

### Influence of clinico-pathological characteristics on *BRAFV600* mutation status

All samples (N = 453) were included in the MLR modeling with main effects only. Age (p = 0.0001), center (p = 0.0004), and melanoma subtype (p = 0.014) significantly influenced *BRAFV600* mutation probability [Table 3], while the influence of UV exposure showed a statistical trend (p = 0.1419). There was no indication that location of sample (p = 0.2966) and nodal status (p = 0.5129) influenced the mutation probability when extended by these two parameters. Furthermore, tumor status, staging, gender, and ulceration did not influence *BRAFV600* mutation probability (all p values >0.4).

**Table 3:** Main effects model with model based p values for the association of *BRAF* mutation status with clinico-pathological factors.

Variable (n = 453)	p value
Age	0.0001
Center	0.0004
Melanoma subtype	0.0140
UV exposure	0.1419

UV, ultraviolet. All model-based p values were determined from Likelihood-Ratio Chi-Square F statistics and adjusted for the influence of all other independent variables in the same model.

**Fig 3:**
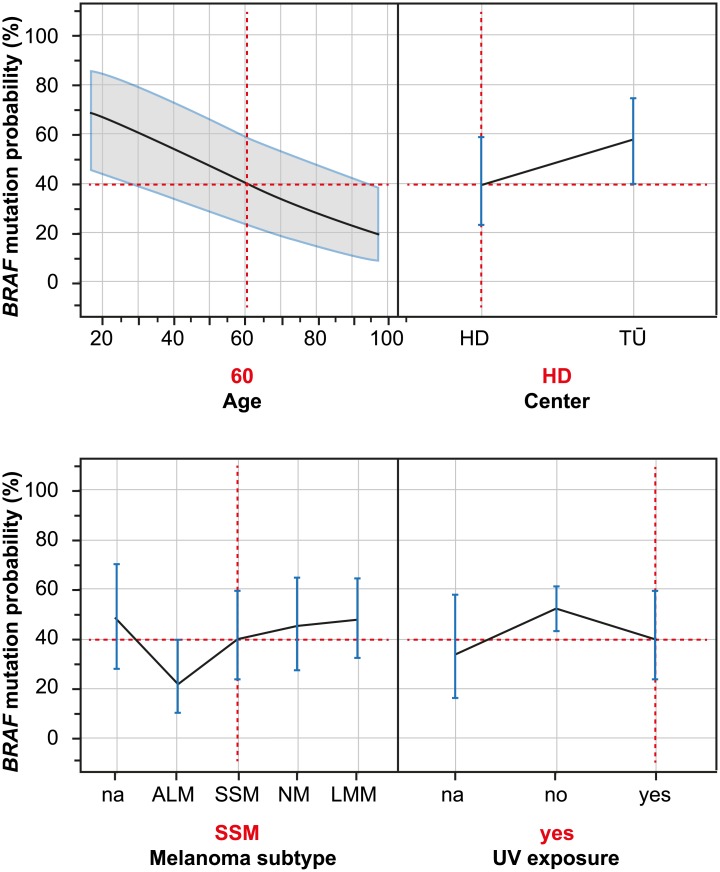
Dependence of the predicted probability of *BRAF* mutation according to the four most influential variables; main effects model. Individual variable effects for each compartment were standardized for the effects of the other three compartments as shown by the vertical dashed red lines. The main effects model allows estimation of the *BRAF* mutation probability for each combination of levels of the four variables: as indicated by the vertical dashed red lines, the estimated *BRAF* mutation probability for a 60-year-old patient at the Heidelberg Center with melanoma subtype SSM and UV exposure was 39.6% (95% confidence interval: 23.1–58.8). The vertical blue lines represent the 95%-confidence intervals, plotted for each level of categorical covariates. In the case of continuous variables, such as age, a 95% confidence interval was plotted (gray). ALM, acral lentiginous melanoma; LMM, lentigo maligna melanoma; NM, nodular melanoma; SSM, superficial spreading melanoma; UV, ultraviolet.

The functional dependence of *BRAF* mutation probability according to the four most influential parameters (age, center, melanoma subtype, and UV exposure) was visualized [Fig 3]. Individual variable effects for each of the four compartments were standardized for the effects of the other three compartments as indicated by the vertical dashed red lines in Fig 3. *BRAF* mutation probability, when estimated by the main effects model, decreased by more than 40% when younger patients were compared with older patients. In the same model, the lowest mutation probabilities were found in patients with ALM and SSM, while mutation probabilities were lower when samples had been obtained from non-UV-exposed regions (versus samples from UV-exposed regions).

In a further analysis step, the interaction between age and the other variables was examined. Samples from 409 patients with available UV exposure data were included in the final MLR interaction model. Center (p = 0.0001) and melanoma subtype (p = 0.0038) significantly influenced *BRAFV600* mutation probability as main effects, while UV exposure (p = 0.0911) alone did not [Table 4]. Interestingly, age no longer had a statistically significant main effect in this model (p = 0.4649), but seemed to be effective only as part of two statistically significant interactions (age x UV exposure [p = 0.0110] and age x melanoma subtype [p = 0.0134]).

**Table 4:** Interaction model for association of *BRAF* mutation status with clinico-pathological factors.

Variable (n = 409)	p value
Center	<0.0001
Melanoma subtype	0.0038
Age x UV exposure	0.0110
Age x melanoma subtype	0.0134
UV exposure	0.0911
Age	0.4649

UV, ultraviolet. All model-based p values were determined from Likelihood-Ratio Chi-Square F statistics and adjusted for the influence of all other independent variables in the same model. x, indicates an interaction between the two corresponding terms allowing that the influence of one variable may depend on the levels of the other variable. In this instance, the different dependence of age from UV exposure or melanoma subtype was indicated.

**Fig 4:**
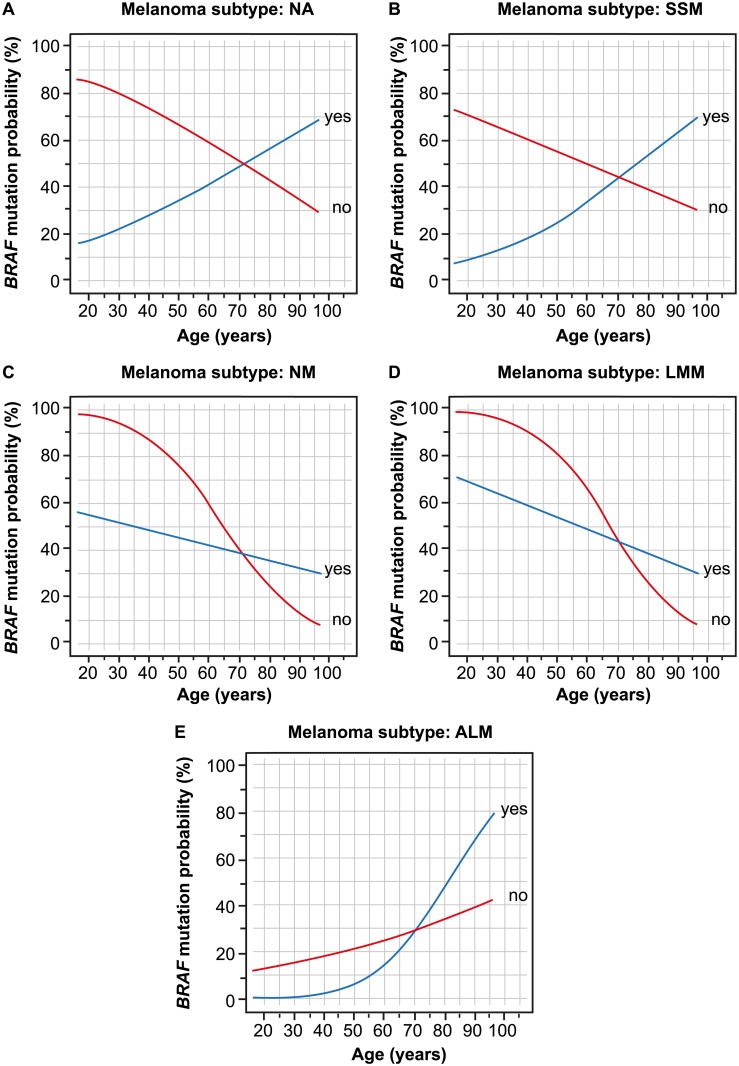
*BRAF* mutation probability changes as a function of age and UV exposure (yes/no). Melanoma subtype was defined as (A) unknown, (B) SSM, (C) NM, (D) LMM, and (E) ALM. Samples were collected from UV exposed area (yes; blue) or non-UV exposed areas (no; red). ALM, acral lentiginous melanoma; LMM, lentigo maligna melanoma; NM, nodular melanoma; SSM, superficial spreading melanoma.

*BRAF* mutation probability varied according to age and UV exposure, depending on melanoma subtype [Fig 4]. In cases of SSM and where melanoma subtype was unknown, similar patterns were observed for the dependency of *BRAF* mutation probability on age and UV exposure. In these two subtypes, *BRAF* mutation probability decreased with age if samples were not in UV-exposed locations; increases were noted in cases with UV exposure (Fig 4a and 4b). For the melanoma subtypes NM and LMM (Fig 4c and 4d), *BRAF* mutation probability decreased with age, although this decrease was weaker if samples were from UV-exposed areas. The pattern observed for ALM (Fig 4e) differed as estimated *BRAF* mutation probability increased with age, although this increase was more pronounced if samples were from UV-exposed areas. There was no significant three-fold interaction between the three parameters.

Analysis of the different *BRAFV600* mutation types (n = 249) found that *BRAFV600E* was significantly influenced by center (p = 0.0020) and gender (p = 0.0386), while location of sample had borderline significance (p = 0.0532). There was no influence of staging. *BRAFV600K* was significantly influenced by location of sample (p = 0.0043) and staging (p = 0.0143).

## Discussion

This study assessed the probability and distribution of *BRAFV600* mutation rates, analyzed using NGS, in patients with melanoma from these two institutes in Germany. Treatment center, melanoma subtype, and age in combination with UV exposure and melanoma subtype all significantly influenced *BRAFV600* mutation probability. The decreasing influence of increasing age on mutation probability was confirmed and quantified. In the main effects model, further investigated in an interaction model, an inverse correlation between age and *BRAFV600* mutation has been reported previously [11, 21]. The sample size of the current study was larger than, or comparable to, those of several other studies reporting *BRAF* mutation rates in melanoma [12–15].

The effect of age on *BRAFV600* mutation probability was significantly less than expected, decreasing or even increasing (p = 0.0110) in primary melanoma cases that were UV-exposed, compared with cases that were not UV-exposed. The effect of age was also dependent on melanoma subtype (p = 0.0134). *BRAFV600K* mutations were influenced by staging and location of sample, with *BRAFV600E* mutations affected by center and gender. The highest probability of *BRAFV600* mutation was noted in patients with LMM. This finding was in line with a previous study that reported a significant difference in *BRAF* mutation frequencies (exon 15) between subtypes (SSM [64.3%], LMM [53.4%], NM [36.4%], and ALM [9.5%]) [22].

The *BRAFV600E;K601I* tandem mutation was detected in 14 patients at the Heidelberg site, but in none at the Tübingen site. This tandem mutation was reported previously in melanoma [23–25] and, while Indsto *et al*. referred to it as a *V600E;K601M* tandem mutation, the base substitution [TG1799_1800AA;A1802T] was the same [23, 25]. The *BRAFV600E;K601I* tandem mutation was verified by four different sequencing methods (two instances of Sanger sequencing, NGS MiSeq System [Illumina, San Diego, CA, USA], pyrosequencing [QIAGEN, Valencia, CA, USA] and GS Junior [NGS] System). In the original report, the patient had metastatic melanoma and a *BRAFV600E* mutation [23]. Due to sensitivity issues we were not able to verify all of the tandem mutations identified with other sequencing methods: rare *BRAFV600* mutations are not detectable with the cobas^®^ 4800 BRAF V600 Mutation Test (Roche Diagnostics, West Sussex, UK) [26], and unfortunately, within the Heidelberg collective the frequency of tandem mutations per sample was too low (<5%) to be detected by pyrosequencing.

This analysis was exploratory and limited, mainly by the differences noted between the two sites. A higher proportion of patients at the Tübingen site had *BRAFV600* mutations, when compared with the Heidelberg site; this difference was both relevant and statistically significant (p = 0.0001). Overall, there were differences in the proportion of mutation frequencies between sites, where Heidelberg had low mutation fractions per sample compared with Tübingen. Heidelberg typically required more tumor tissue than Tübingen for routine diagnostics, including immunohistochemistry staining for melanoma diagnosis, resulting in less availability of tumor material for further analysis. Thus, the reason for the observed differences in *BRAFV600* mutation probability was likely to be technical. There were also more patients with primary melanoma at the Heidelberg site (p<0.0001), although UV exposure was balanced between sites.

The ability to predict *BRAFV600* mutation probability accurately based on covariates may be beneficial in cases where tumor tissue is not available or where treatment is time-limited, e.g. in the case of patients with life-threatening metastases. We generated a model providing predicted *BRAFV600* positivity probabilities for a comprehensive set of combinations of covariate factor levels. This is not intended to replace *BRAFV600* testing by pathological institutes, but to provide support as a tool for standardization and quality control. Further validation of the model in a multicenter study with documentation of UV exposure is required to confirm reproducibility and applicability for clinical use.

## Conclusions

In conclusion, this study reported on the patient- and tumor-related covariates that may impact *BRAF* mutation probability in patients with melanoma across two treatment centers in Germany. Additional validation of our statistical model is required; however, identification of underlying *BRAF* mutations is crucial for selection of appropriate therapy.

## Supporting information

S1 Table *BRAF* mutation type by center. (DOCX)
