# Supplementary material for: Variables that influence BRAF mutation probability: A next-generation sequencing, non-interventional investigation of BRAFV600 mutation status in melanoma
Source: PLoS One. 2017 Nov 27;12(11):e0188602. doi: 10.1371/journal.pone.0188602 (PMC5703505; doi:10.1371/journal.pone.0188602)
Supplement: S1 Table — (DOCX) [file pone.0188602.s001.docx]

**S1 Table. *BRAF* mutation type by center.**

| *BRAF* mutation type | Center | | | | | |
| --- | --- | --- | --- | --- | --- | --- |
|  | Heidelberg | | Tübingen | | All | |
|  | n | % | n | % | n | % |
| K601E | 2 | 0.9 | 3 | 1.2 | 5 | 1.1 |
| K601R | 1 | 0.5 | 0 | 0 | 1 | 0.2 |
| L597Q | 1 | 0.5 | 0 | 0 | 1 | 0.2 |
| S605N | 1 | 0.5 | 0 | 0 | 1 | 0.2 |
| S605S | 1 | 0.5 | 0 | 0 | 1 | 0.2 |
| S607P | 1 | 0.5 | 0 | 0 | 1 | 0.2 |
| V600D | 0 | 0 | 2 | 0.8 | 2 | 0.4 |
| V600E | 66 | 31.1 | 135 | 56.0 | 201 | 44.4 |
| V600E2 | 11 | 5.2 | 3 | 1.2 | 14 | 3.1 |
| V600E2;K601I | 14 | 6.6 | 0 | 0 | 14 | 3.1 |
| V600G | 0 | 0 | 1 | 0.4 | 1 | 0.2 |
| V600K | 4 | 1.9 | 12 | 5.0 | 16 | 3.5 |
| V600M | 0 | 0 | 3 | 1.2 | 3 | 0.7 |
| WT | 110 | 51.9 | 82 | 34.0 | 192 | 42.4 |
| All | 212 | 100 | 241 | 100 | 453 | 100 |
